# Novel mutation of the *LPIN2* gene in Turkish brothers with Majeed syndrome. Response to IL-1 inhibition

**DOI:** 10.1186/1546-0096-9-S1-P300

**Published:** 2011-09-14

**Authors:** T Herlin, M Bjerre, B Fiirgaard, G Kerndrup, H Hasle, P Ferguson

**Affiliations:** 1 Department of Pediatrics, Aarhus University Hospital Skejby; 2 Clinical Research Laboratory, Aarhus University Hospital, Vejle Hospital, Denmark; 3 The MR Research Centre, Aarhus University Hospital Skejby; 4 Department of Clinical Genetics, Vejle Hospital, Denmark; 5 Department of Pediatrics, University of Iowa Carver College of Medicine, Iowa, USA

## Background

Majeed syndrome is a rare, syndromic form of chronic recurrent multifocal osteomyelitis (CRMO) first described in 1989. The syndrome starts during infancy with recurrent relapses of osteomyelitis typically associated with fever, congenital dyserythropoietic anemia (CDA) and often neutrophilic dermatosis (Sweet syndrome). Homozygous mutations in the *LPIN2* gene located on the short arm of chromosome 18 have been identified as being responsible for the Majeed syndrome.

## Aim

To report a novel mutation in the *LPIN2* gene detected in two brothers with Majeed syndrome and to describe the clinical characteristics and response to treatment.

## Case presentations

Two Turkish brothers (13 (Y) and 29 (M) months old) with consanguinity of the parents were admitted with relapsing episodes of pain and ‘pseudoparalysis’ of upper and lower extremities since the age of 3 and 6 months, respectively. No concomitant fever has occurred during the attacks. Whole body MRI of the elder brother (M) revealed osteomyelitic changes of the metaphyses of tibiae, left fibula and left radius. Biopsy from lesions showed no malignancy and negative bacterial cultures. Both showed significant hypersedimentation (ESR 92 mm/hr (Y) and 96 mm/hr (M)), slight thrombocytosis, and moderate anemia (Hb 9.0 (Y) and 9.7 (M) g/dl). Bone marrow aspiration was consistent with congenital dyserythropoietic anemia (CDA) with 6% - 9% bi- or multinucleated erythrocytes. *LPIN2* gene re-sequencing of each affected child revealed a homozygous 2 bp deletion (c.1312_1313delCT) resulting in an early truncation of the protein (L438fs+16X), which confirmed the diagnosis in both patients. Clinically, both were refractory to the treatment with corticosteroids and TNFα inhibition (etanercept). Elevated plasma levels of proinflammatory cytokines (IL-1β, IL-6, IL-8, TNFα) did not change significantly during the treatment. In M, rapid clinical and laboratory improvement was observed after introduction with anakinra (1.7 mg/kg/d), which has not yet been introduced to Y.

## Conclusion

We describe a novel mutation of the *LPIN2* gene in two Turkish brothers with Majeed syndrome. Although our patients also presented with CDA none of them had fever during the attacks nor dermatological changes unlike previously described patients with Majeed. IL-1 inhibition showed promising clinical and laboratory results.

